# Comparative transcriptome reveals the potential modulation mechanisms of estradiol affecting ovarian development of female *Portunus trituberculatus*

**DOI:** 10.1371/journal.pone.0226698

**Published:** 2019-12-19

**Authors:** Meimei Liu, Jie Pan, Zhiguo Dong, Yongxu Cheng, Jie Gong, Xugan Wu

**Affiliations:** 1 Key Laboratory of Exploration and Utilization of Aquatic Genetic Resources, Ministry of Education, Shanghai Ocean University, Shanghai, China; 2 Key Laboratory of Marine Biotechnology of Jiangsu Province, Huaihai Institute of Technology, Lianyungang, China; 3 National Demonstration Centre for Experimental Fisheries Science Education, Shanghai Ocean University, Shanghai, China; 4 Centre for Research on Environmental Ecology and Fish Nutrition of Ministry of Agriculture, Shanghai Ocean University, Shanghai, China; 5 School of Life Sciences, Nantong University, Nantong, China; Zhejiang University College of Life Sciences, CHINA

## Abstract

Estradiol is an important sex steroid hormone that is involved in the regulation of crustacean ovarian development. However, the molecular regulatory mechanisms of estradiol on ovarian development are largely unknown. This study performed transcriptome sequencing of ovary, hepatopancreas, brain ganglion, eyestalk, and mandibular organ of crabs after estradiol treatment (0.1μg g^-1^ crab weight). A total of 23, 806 genes were annotated, and 316, 1300, 669, 142, 383 genes were expressed differently in ovary, hepatopancreas, brain ganglion, eyestalk, and mandibular organ respectively. Differentially expressed gene enrichment analysis revealed several crucial pathways including protein digestion and absorption, pancreatic secretion, insect hormone biosynthesis, drug metabolism-cytochrome P450 and signal transduction pathway. Through this study, some key genes in correlation with the ovarian development and nutrition metabolism were significantly affected by estradiol, such as *vitelline membrane outer layer 1-like protein*, *heat shock protein 70*, *Wnt5*, *JHE-like carboxylesterase 1*, *cytochrome P302a1*, *crustacean hyperglycemic hormone*, *neuropeptide F2*, *trypsin*, *carboxypeptidase B*, *pancreatic triacylglycerol lipase-like*, and *lipid storage droplet protein*. Moreover, RT-qPCR validation demonstrated that expression of transcripts related to ovarian development (*vitelline membrane outer layer 1-like protein* and *cytochrome P302a1*) and nutrition metabolism (*trypsin*, *glucose dehydrogenase* and *lipid storage droplet protein*) were significantly affected by estradiol treatment. This study not only has identified relevant genes and several pathways that are involved in estradiol regulation on ovarian development of *P*. *trituberculatus*, but also provided new insight into the understanding of the molecular function mechanisms of estradiol in crustacean.

## Introduction

In many decapod crustacean, regulation of reproductive maturation is a major process in commercial aquaculture. Reproduction in female crustaceans is characterized by ovarian development, which includes the two processes of oogenesis and vitellogenesis [1, 2]. Crustacean ovarian development has been proved to be regulated by various hormonal factors [3]. Estradiol, an important sex steroid, is an important active estrogen in most crustacean, which promotes the process of vitellogenesis and ovarian development [4–6]. However, the negative effects of estradiol on ovarian development and vitellogenesis were not detected on some crustaceans with a particular ovarian stage, including the immature tiger prawn, *Penaeus esculentus* and ridgeback shrimp *Sicyonia ingentis*, during the non-reproductive stage [7, 8]. The possible reason for the contradictory results was that the effects of estradiol on vitellogenesis are ovarian stage-specific and dose-dependent for crustaceans [5, 9, 10]. Furthermore, previous studies have shown that estradiol is distributed widely in several tissues such as hepatopancreas, ovary and hemolymph, and the concentration of estradiol shows a significant positive correlation with the ovarian development [11–13]. However, the molecular regulatory mechanisms of estradiol on ovarian development are largely unknown in crustacean. Transcriptome sequencing enables the production of high-throughput fragments of double-stranded cDNA and the rapid assembly of sequences for annotation. It facilitates gene discovery and broadens our understanding of gene networks, especially in non-model organisms with unknown genomes [14].

Over the past few decades, numerous studies have shown that the endocrine regulation of crustacean ovarian development is complex with multiple hormonal factors employed to positively control ovarian development [3, 15, 16]. For example, X-organ-sinus gland complex system in the eyestalk secretes many crucial neuropeptide hormones, including crustacean hyperglycemic hormone and molt inhibiting hormone that exhibit a positive influence on vitellogenin activities [17–19]. Brain ganglion secretes biogenic amines and gonad stimulatory hormone could also promote the vitellogenesis of crustacean, and biogenic amines can enhance the release of gonad stimulatory hormone [20]. Similarly, methyl farnesoate and ecdysteroids secreted by mandibular organ and Y-organ, respectively, also exhibited gonad stimulatory function [3, 15, 21, 22]. Remarkably, vertebrate-type sex steroids, especially estrogen and progesterone, play a vital role in crustacean vitellogenesis by stimulating related metabolic pathways initiation during vitellogenesis, such as lipogenesis [15]. Those hormonal factors originating from different endocrine organs are involved in ovarian development of crustaceans individually or in synergy with one another, but data on this aspect are still inadequate [3].

The swimming crab, *Portunus trituberculatus*, is an important marine-culture crab widely distributed in the coastal water area of East Asia, including Korea, Japan, Philippines and China [23, 24]. Previous studies in our group have shown that estradiol widely distributed in various endocrine organs of *P*. *trituberculatus* and exogenous estradiol could promote the vitellogenesis and ovarian development [10, 25, 26]. Therefore, there are two objectives in this study: (1) to obtain ten reference transcriptome assemblies for the ovary, hepatopancreas, brain ganglion, eyestalk and mandibular organ from control crabs and those exposed to an acute level of estradiol; (2) to identify estradiol-responsive gene profile differences to unravel the different mechanisms of action of estradiol in five organs. The results will provide new insight into the understanding of the molecular regulatory mechanisms of estradiol in *P*. *trituberculatus* and other homologous species.

## Materials and methods

### Experimental animals, design, and sampling

Female *P*. *trituberculatus* that have just finished puberty molt (body weight = 150 ± 25 g) were collected from outdoor ponds of Qidong scientific research base, Shanghai Fisheries Research Institute, Jiangsu, China, and then acclimated for a week in the indoor circulating water system. During the experiment, these crabs were individually cultured in the culture box (L × W × D = 33 × 27.5 × 35 cm), all culture boxes were floating in two concrete tanks (Length × Width × Depth = 5.8 m × 2.4 m × 1.8 m) by foam attached to the surrounding of the boxes. There were many uniform holes on the walls of each box that allow ample water exchange with the concrete tanks. The water depth of each basket was maintained at ca. 25 cm with a layer of 5–6 cm sand provided to the bottom of each boxes for the crabs to bury [27]. Prior to beginning the experiment, 20 crabs were randomly divided into control (n = 10) and treatment groups (n = 10). The crabs assigned to the treatment group received estradiol solution (Sigma-Aldrich, dissolved in absolute ethanol) injection through the arthrodial membrane at the base of the swimming-leg. The injection dose (0.1μg g^-1^ crab weight) and volume (0.5μl g^-1^ crab weight) were followed the previous publications on the other decapod crustacean [4, 7, 28]. The control crabs received the same volume of absolute ethanol [4, 7, 28]. 24 h post injection, eight crabs from each group were randomly sampled for RNA extraction and RNA-seq analysis. All samples, including the ovary (O), hepatopancreas (H), brain ganglion (BG), eyestalk (SG) and mandibular organ (MO), were frozen with liquid nitrogen and then stored at -80°C until analysis. All crabs were treated in strict accordance with the guidelines for the care and use of experimental animals established by the Administration of Affairs Concerning Experimental Animals of the State Council of the People’s Republic of China, and approved by the Committee on Experimental Animal Management of the Shanghai Ocean University.

### RNA extraction, cDNA library preparation and next-generation sequencing

Total RNA was extracted from the tissues of *P*. *trituberculatus* using Trizol Reagent (Takara) according to the manufacturer's recommendations and genomic DNA was removed with DNase I (Takara). Subsequently, the integrity and purity were estimated by a 2100 Bioanalyzer (Agilent Technologies, USA) and NanoDrop 2000 (Thermo Fisher Scientific Inc., USA), respectively. Only high-quality RNA samples (OD260/280 ranged 1.8–2.2, RIN ≥8.0) were used to construct the sequencing library.

The cDNA libraries were generated using the TruSeq RNA sample prep kit (Illumina) following manufacturer's recommendations. The synthesized cDNA libraries were checked using PicoGreen (Quantifluor^™^-ST fluorometerE6090, Promega, CA, USA) and fluorospectrophotometry (Quant-iT PicoGreen dsDNA Assay Kit; Invitrogen, P7589) and quantified with Agilent 2100 (Agilent 2100 Bioanalyzer, Agilent, 2100; Agilent High Sensitivity DNA Kit, Agilent, 5067–4626). The final sequencing cDNA libraries were quantified to 4–5 pM and sequenced using the Illumina HiSeq 4000 with 150 bp pair-end reads produced (Illumina, USA).

### *De novo* assembly and annotation functional of the transcriptome

Before proceeding to *de novo* assembly, the raw reads were first quality-filtered to remove adaptor sequences, ambiguous ‘N’ nucleotides (with an ‘N’ ratio over 10%), and low-quality sequences (with quality scores lower than 20). The clean reads were assembled into non-redundant transcripts using the Trinity software, which has been developed specifically for the *de novo* assembly of transcriptomes using short reads [29]. The assembled unigenes were then used for sequence annotation using the databases including the NCBI non-redundant protein database (NR), Gene Ontology (GO), Evolutionary genealogy of genes: Non-supervised Orthologous Groups (eggNOG), Swiss-Prot protein, and Kyoto Encyclopedia of Genes and Genome (KEGG) [30–32].

### Identification of differentially expressed genes (DEGs)

The expression of all unigenes was estimated by calculating read density as ‘reads per kb per million reads’ (RPKM) using the RSEM program [33]. To identify the DEGs, |log2(FoldChange)| > 1 & P-value < 0.05 were set to be the threshold for significantly different expression levels. The gene expression profiles were compared with estradiol and control group, and then all DEGs in each comparison were carried on the GO functional and KEGG pathway enrichment analysis using GO database and KEGG database.

### Validation of the RNA-seq profiles by quantitative real-time PCR (qPCR)

Randomly selected DEGs identified by transcriptome sequencing analysis were used for RNA-seq validation by qRT-PCR. RNA from 24h samples (8 replicates from each treatment) was used for cDNA synthesis using a reverse first strand cDNA synthesis kit (RR036A, Takara Bio, Japan). The qRT-PCR assays were performed with three replicates, and the 18S gene was used as an internal control to normalize the expression level of the target genes [34]. The specific primers were designed according to the unigene sequences using Primer 5.0 software (**Table 1**).

**Table 1:** Specific primers used to in qRT-PCR.

Primer name	Sequence (5′→3′)
Vitelline membrane outer layer 1-like protein	F: *GTTCGAGCCCTACACCTTCTTG* R: *ACGATCTCATGTCCTGCCACTC*
Glucose dehydrogenase	F: *TATGATGCCGCATGGATTTCTG* R: *TCCTTGTGGACGGGTAGATGAC*
Lipid storage droplet protein	F: *GCCGTTCCGATAATCAACCTAC* R: *AGAGTTTGGGGCACGATCAAGT*
CYP302a1	F: *CCAACTGTCTTGTTCTCCCCACT* R: *CTCTTCTGCCGAGCATGTCTCA*
Trypsin	F: *GACAGCACGGCACTTACAAACG* R: *TACGATGCAATCAACGCCTACA*
18S	F: *TCCAGTTCGCAGCTTCTTCTT* R: *AACATCTAAGGGCATCACAGACC*

The volume of the qRT-PCR reaction system was 10 μl, containing 5 μL of 2×SYBR Master Mix (RR420A, Takara Bio, Japan), 0.2 μL of each primer and ROX Reference Dye II (RR420A, Takara Bio, Japan), 1 μL of cDNA template, and 3.4 μl of RNase Free dH_2_O. The qPCR was carried out in a FAST-7500 system (ABI-7500, ThermoFisher, Singapore) as follows: 95°C for 30 s; 40 cycles of 95°C for 5 s, 60°C for 30 s, and 72°C for 30 s. The relative quantification of qRT-PCR data was calculated using the 2^-ΔΔCt^ method [35]. Data were presented as the mean ± standard error (SE). All statistical analyses were performed using SPSS 19.0 software (SPSS, Chicago, USA).

## Results

### Sequencing and *de novo* assembly

A total of 441,394,570 Illumina HiSeq reads from ovary, hepatopancreas, brain ganglion, eyestalk and mandibular organ were generated. Each sample yielded more than 40 million reads of data. All raw reads and quality control statistics are presented in **Table 2**.

**Table 2:** Raw reads and quality control of reads for *P*. *trituberculatus* libraries.

Tissue	Treatment	Number of raw reads	GC (%)	Q30 (%)	Number of clean reads	Reads filtered (%)
Ovary	estradiol (O-1)	45,496,138	56.87	93.84	44,982,290	98.87
	ethanol (O-2)	47,538,614	56.64	93.29	46,931,094	98.72
Hepatopancreas	estradiol (H-1)	42,206,722	57.64	93.59	41,865,022	99.19
	ethanol (H-2)	43,020,694	55.87	93.95	42,674,458	99.19
Brain ganglion	estradiol (BG-1)	43,396,114	53.85	93.3	43,007.618	99.1
	ethanol (BG-2)	47,549,836	54.45	93.42	47.161,352	99.18
Eyestalk	estradiol (SG-1)	44,105,484	55.89	93.52	43,742,076	99.17
	ethanol (SG-2)	45,100,746	56.2	93.81	44,768,352	99.26
Mandibular organ	estradiol (MO-1)	42,104,066	56.65	92.81	41,865,022	99.06
	ethanol (MO-2)	40,876,156	57.69	93.26	40,621,404	99.37

*De novo* sequence assembly of the current dataset using the Trinity assembler generated 106,130 transcripts, with an average transcript size of 681 base pairs (bp) and N50 of 1070 bp. Finally, 84, 032 unigenes were obtained after combining the transcripts, with a total length of 49,104,718 bp. The mean unigene length was 584.36 bp, and the N50 was 789 bp. A brief summary of *de novo* assembly statistics was provided in **Table 3**. The raw data were uploaded to the National Center for Biotechnology Information (NCBI, PRJNA532740).

**Table 3:** De novo assembly statistics of the *P*. *trituberculatus* transcriptome.

	Transcripts	Unigenes
Total number	106,130	84,032
Total length	72,377,107	49,104,718
Max. length (bp)	14,331	14,331
Mean length (bp)	681.97	584.36
N50 length (bp)	1,070	789
GC%	50.32	59.36

### Functional annotation

**Fig 1:**
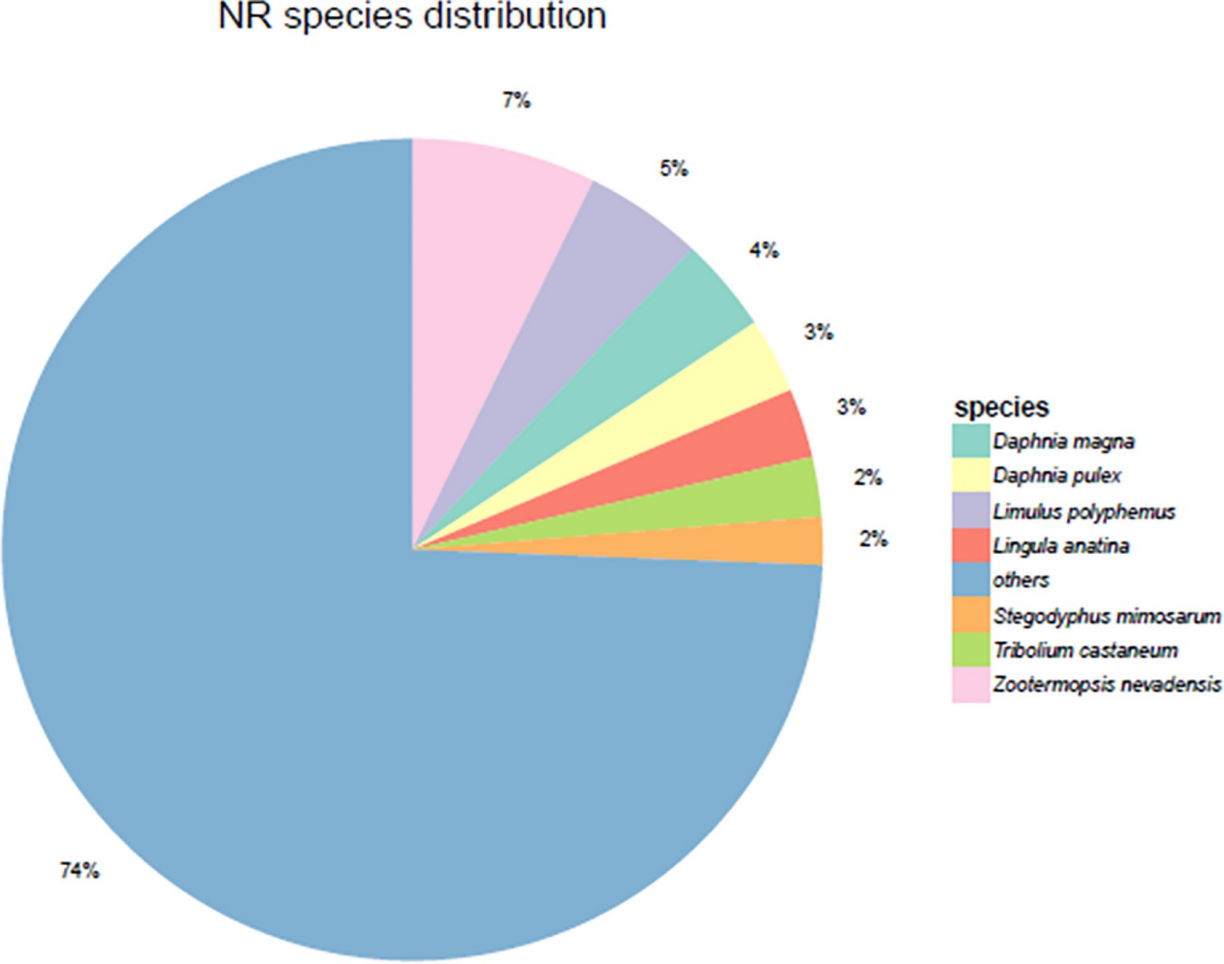
Species distribution of BLASTx hits.

Functional annotation of the assembly identified 84,033 unique protein-encoding genes, 23,806 (28.33%) of which could be matched to the NR database (**Table 4**), while 9, 650 (11.48%) transcripts could be fully annotated with GO available data, illustrating the scarcity of crustacean sequences in current genomic databases. Species most represented in the BLASTx searches included *Zootermopsis nevadensis* (7%), *Limulus polyphemus* (5%) and *Daphnia magna* (4%), but “other” species was the largest group (74%) (**Fig 1**).

**Table 4:** Summary of functional annotation of *P*. *trituberculatus* transcriptome.

Annotation in Database	Unigene No.	Percentage (%)
NR	23,806	28.33
GO	9,650	11.48
KEGG	5,814	6.92
eggNOG	22,625	26.92
Swissprot	21,439	25.51

**Fig 2:**
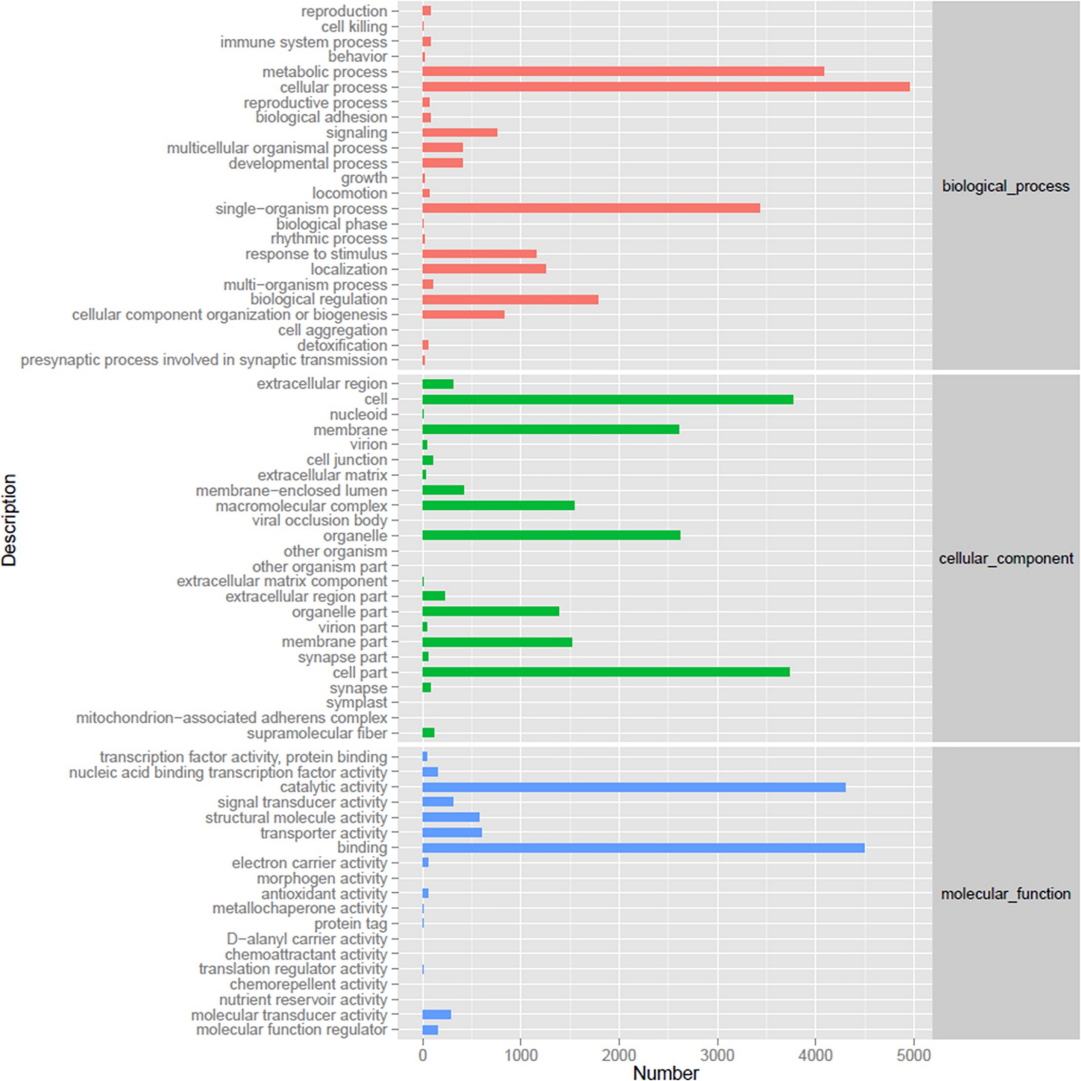
Gene Ontology (GO) categorization for assembled unigenes.

The GO assignment programs were also used for functional categorizations of the annotated unigenes. These unigenes were classified into three groups including biological processes, cellular components, and molecular function. These unigenes were then further divided into 67 functional subgroups. Among biological processes category, metabolic process (4,079) and cellular process (4,950) were the most enriched GO terms. Within the cellular components, transcripts assigned to cell (3,762), cell part (3,734), and organelle (2,622) were the three most abundant. In molecular function category, binding-related genes (4,498) and catalytic activity (4,303) were the most enriched GO terms (**Fig 2**).

**Fig 3:**
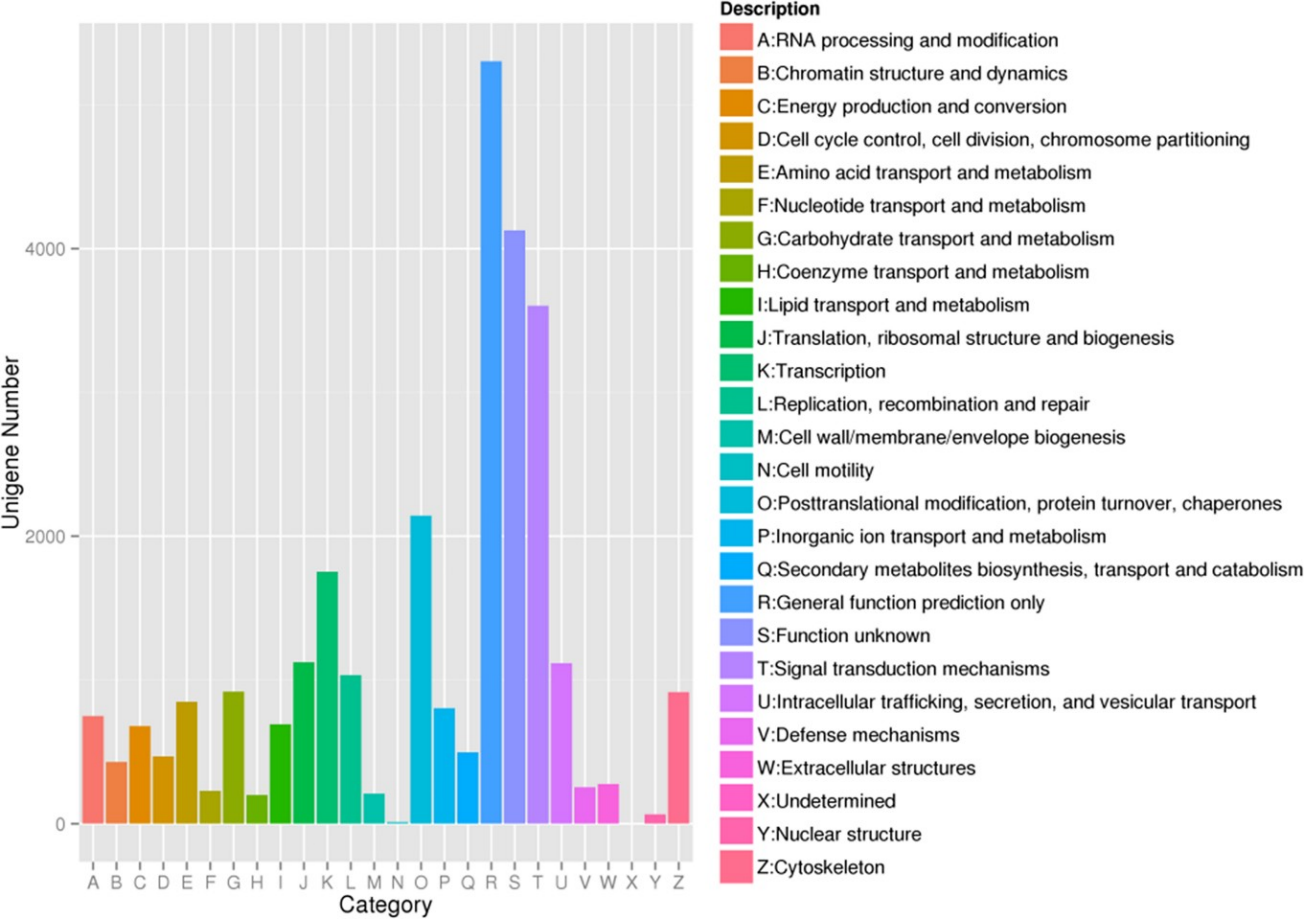
Clusters of eggNOG functional classification of *P*. *trituberculatus* transcriptome.

Assignments of eggNOG were further used to evaluate the completeness of the transcriptome library and the efficiency of the annotation process. These sequences were categorized into 25 categories. Of these, the “general function prediction only” (5,303) was the largest cluster and “Cell motility” (12) was the smallest one (**Fig 3**).

**Fig 4:**
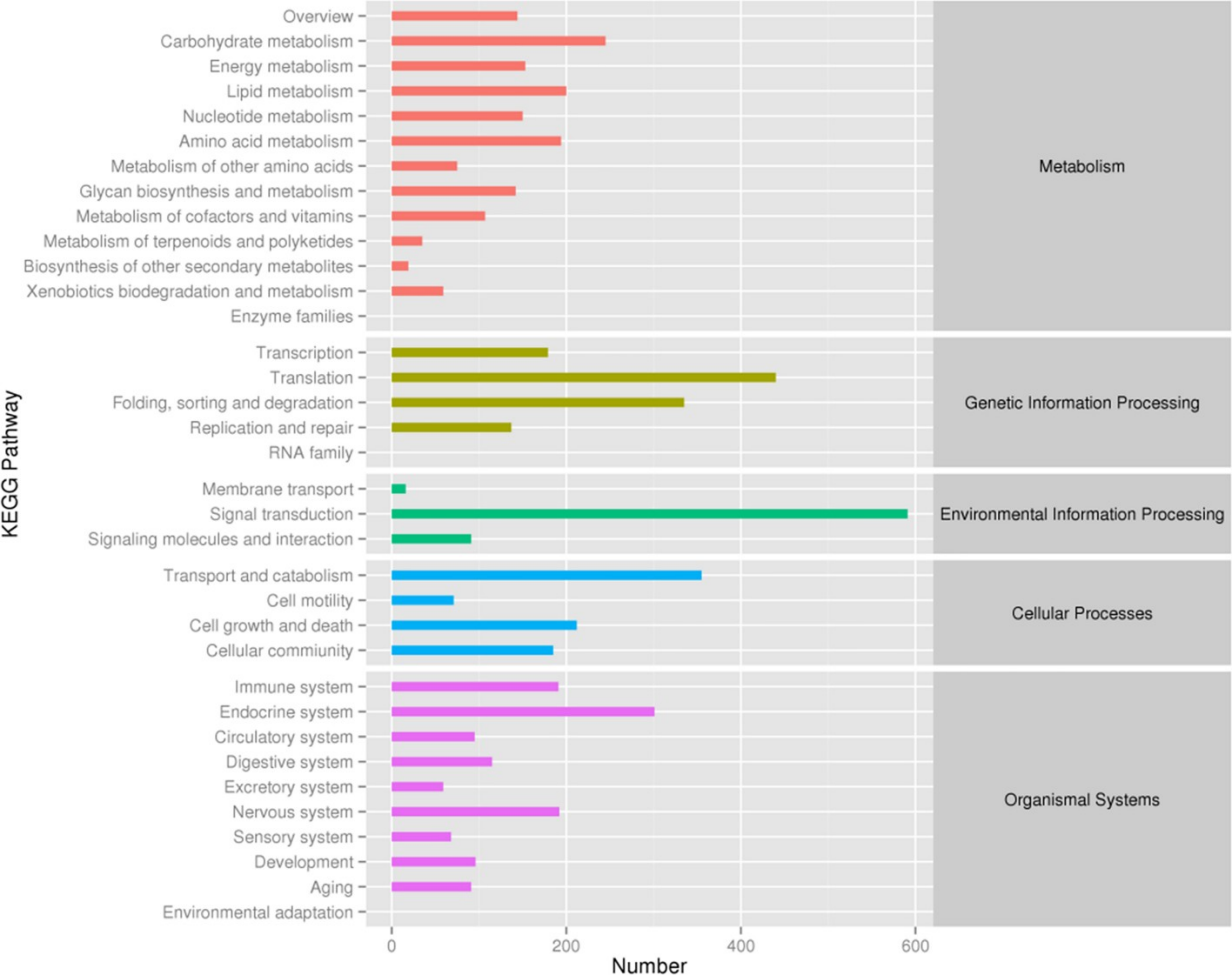
Distribution of unigene numbers for the major KEGG pathway categories in the *P*. *trituberculatus* transcriptome.

To systematically analyse the associated intracellular and intercellular metabolic pathways as well as complicated biological behaviours, the unigenes were annotated by the KEGG database. A total of 5,814 unigenes were assigned to five pathway categories, including metabolism, genetic information processing, environmental information processing, cellular processes and organismal systems, and further predicted in 33 specific pathways (**Fig 4**). The largest pathway group was environmental information processing in the “signal transduction”, which contained 591 genes.

### Differentially expressed genes (DEGs)

In the current study, the DEGs were selected according to the criteria of a P value ≤ 0.05 and a |log2(FoldChange)| > 1, and the clustering of transcripts according to patterns of differential expression across samples (**Table 5**). For the ovary, 316 DEGs were detected between the estradiol and control groups, of which 76 and 240 unigenes were up-regulated and down-regulated respectively. For the hepatopancreas, 1300 DEGs were detected between the estradiol and control groups, of which 1191 and 109 unigenes were up-regulated and down-regulated respectively. For the, brain ganglion, 669 DEGs were detected between the estradiol and control groups, of which 268 and 401 unigenes were up-regulated and down-regulated respectively. For the eyestalk, 142 DEGs were detected between the estradiol and control groups, of which 21 and 121 unigenes were up-regulated and down-regulated respectively. For the mandibular organ, 383 DEGs were detected between the estradiol and control groups, of which 233 and 150 unigenes were up-regulated and down-regulated respectively.

**Table 5:** Summary statistics of differentially expressed genes.

DEG Set	DEG Number	Up-regulated	Down-regulated
O-1 vs O-2	316	76	240
H-1 vs H-2	1300	1191	109
BG-1 vs BG-2	669	268	401
SG-1 vs SG-2	142	21	121
MO-1 vs MO-2	383	233	150

### GO and KEGG enrichment analysis of DEGs

To further assign the putative functions to DEGs, we performed GO and KEGG pathway analyses.

**Fig 5:**
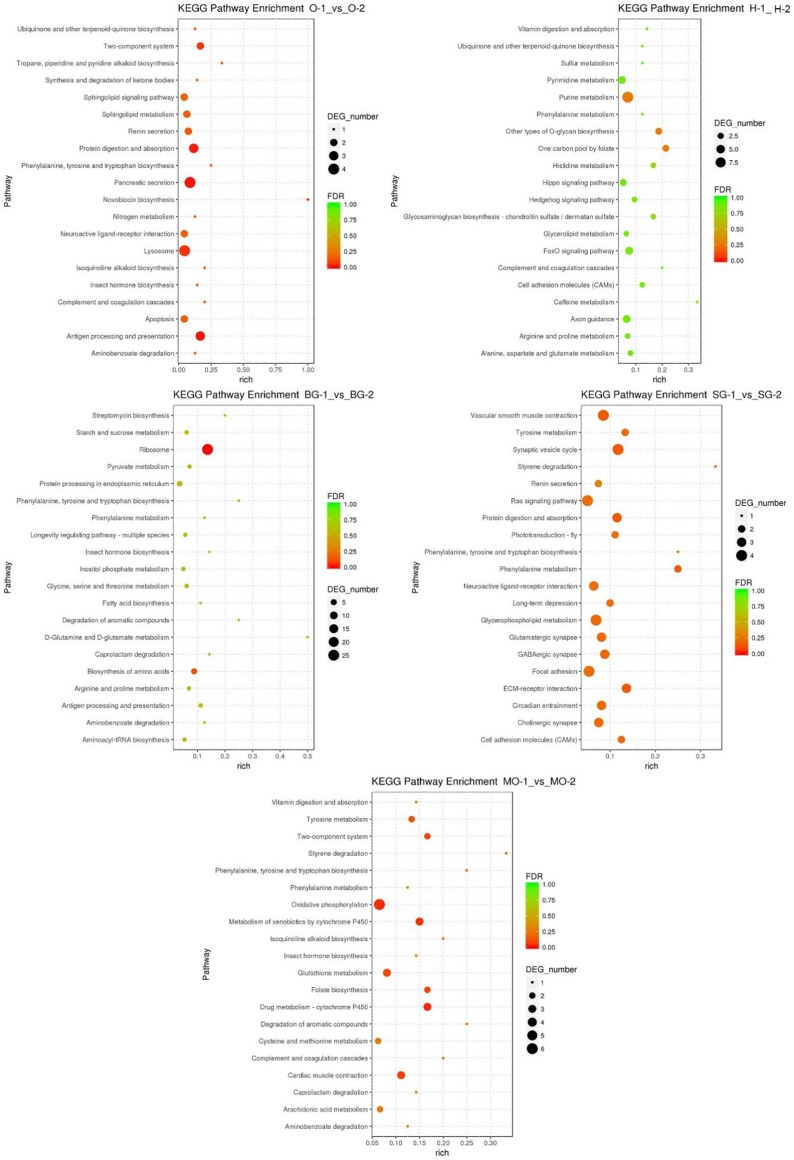
KEGG enrichment analyses of DEGs. The enrichment factor indicates the ratio of the DEGs number to the total gene number in a certain pathway. The size and color of the dots represent the gene number and the range of P values, respectively. The 1 and 2 represent estradiol group and control group, respectively. O: ovary; H: hepatopancreas; BG: brain ganglion; SG: eyestalk; MO: mandibular organ.

The DEGs were assigned to 205, 488, 337, 441, and 319 GO terms in the ovary, hepatopancreas, brain ganglion, eyestalk and mandibular organ, respectively. The most significantly enriched top 20 metabolic pathways were represented for each tissue between estradiol and control group (**Fig 5**). In the ovary, the significantly enriched pathways included “Antigen processing and presentation”, “Pancreatic secretion” and “Protein digestion and absorption”. In the hepatopancreas, the significantly enriched pathways were “Purine metabolism” and “Other types of O-glycan biosynthesis”. The most significantly enriched pathways for the brain ganglion were “Ribosome-genetic information processing” and “Biosynthesis of amino acids”. The DEGs in eyestalk were significantly clustered in “Vascular smooth muscle contraction” and “Synaptic vesicle cycle”. For the mandibular organ, the significantly enriched pathways included “Oxidative phosphorylation”, “Drug metabolism-cytochrome P450” and “Metabolism of xenobiotics by cytochrome P450”.

### Key genes in response to estradiol treatment

Since the proteins and lipids are the main constituents of yolk, the process of nutrients metabolism is active during the period of ovarian development. Among the genes that were found to be differentially expressed in the estradiol-injection crab compared to the control, several candidate genes that were thought to play an important role in crustacean ovarian development and nutrient metabolism were further identified (**Table 6**). In the ovary, the *vitelline membrane outer layer 1-like protein*, *carboxypeptidase B*, *trypsin-like serine proteinase* and *trypsin* were down-regulated by estradiol injection. In the hepatopancreas, estradiol injection up-regulated the expression levels of 5 genes (*pancreatic triacylglycerol lipase-like*, *very low-density lipoprotein receptor*, *fatty acid elongase protein*, *alcohol dehydrogenase and lipid storage droplet protein*) involved in the metabolic process. For genes involved in reproductive process in hepatopancreas, the transcripts of 9 genes significantly increased in estradiol treatment, while the expression level of *heat shock protein 70* significantly decreased. In brain ganglion, estradiol injection up-regulated the expression levels of *cyclin B*, *JHE-like carboxylesterase 2 and juvenile hormone esterase-like protein*, while down-regulated the transcripts of *Cytochrome P 302a1*. In mandibular organ, the expression levels of *JHE-like carboxylesterase 1*, *juvenile hormone acid methyltransferase and C-type allatostatin* significantly decreased in estradiol treatment. Interestingly, 4 genes (*crustacean hyperglycemic hormone 1*, *molt-inhibiting hormone*, *neuropeptide F2 and SIFamide*) involved in reproductive process in eyestalk were up-regulated under estradiol injection. In addition, estradiol significantly altered the expression levels of a novel transcript encoding *ovary development-related protein*.

**Table 6:** Summary statistics of differentially expressed genes.

Functional category	Gene id	Tissue	Full name	FC^a^	P-value
**Vitellogenesis**					
	c89341_g1	Ovary	*Vitelline membrane outer layer 1-like protein*	0.25	7.55E-03
**Reproduction**					
	c112734_g3	Hepatopancreas	*Heat shock protein 70*	0.009	3.67E-04
	c111383_g1	Hepatopancreas	*Vasa-like protein*	485.84	2.38E-03
	c93926_g2	Hepatopancreas	*Wnt-5b*	Inf	2.41E-02
	c120259_g1	Hepatopancreas	*Wnt5a ligand*	Inf	3.85E-02
	c116484_g2	Hepatopancreas	*Insulin-like receptor*	Inf	4.42E-04
	c118618_g1	Hepatopancreas	*Neuropeptide receptor B3*	28.87	9.09E-03
	c115847_g1	Hepatopancreas	*Putative cytoplasmic polyadenylation element-binding protein*	243.78	5.73E-05
	*c113803_g1*	Brain ganglion	*Cyclin B*	2.90	*2*.*07E-02*
	c108894_g1	Hepatopancreas	*Ovary development-related protein*	156.86	2.69E-04
	c121312_g1	Hepatopancreas	*JHE-like carboxylesterase 1*	21.23	8.08E-03
	*c62034_g1*	Brain ganglion	*JHE-like carboxylesterase 2*	282.21	*3*.*07E-09*
	*c118386_g3*	Mandibular organ	*JHE-like carboxylesterase 1*	0.12	*1*.*17E-04*
	c92476_g1	Brain ganglion	*Juvenile hormone esterase-like protein*	2.32	1.56E-02
	*c108242_g2*	Mandibular organ	*Juvenile hormone acid methyltransferase*	0.33	*3*.*03E-02*
	c111705_g1	Hepatopancreas	*Estrogen sulfotransferase*	343.62	3.03E-05
	*c83592_g2*	Brain ganglion	*Cytochrome P 302a1*	0.48	*3*.*87E-02*
	*c15627_g1*	Eyestalk	*Crustacean hyperglycemic hormone 1*	210.38	*1*.*26E-07*
	*c87010_g1*	Eyestalk	*Molt-inhibiting hormone*	1823	*3*.*79E-09*
	*c82338_g1*	Eyestalk	*Neuropeptide F2*	49.75	6.54E-04
	*c100581_g1*	Eyestalk	*SIFamide*	24.25	*4*.*17E-04*
	*c100697_g1*	Mandibular organ	*C-type allatostatin*	0.08	2.85E-03
**Protein metabolism**					
	c120409_g1	Ovary	*Carboxypeptidase B*	0.12	1.07E-20
	c110626_g1	Ovary	*Trypsin*	0.15	2.64E-19
	c115002_g1	Ovary	*Trypsin-like serine proteinase*	0.16	2.80E-16
**Lipid metabolism**					
	c116241_g1	Hepatopancreas	*Pancreatic triacylglycerol lipase-like*	Inf	1.06E-02
	c96963_g1	Hepatopancreas	*Very low-density lipoprotein receptor*	Inf	4.71E-03
	c60052_g1	Hepatopancreas	*Fatty acid elongase protein*	Inf	1.82E-02
	c110929_g1	Hepatopancreas	*Alcohol dehydrogenase*	Inf	1.21E-04
	c118072_g2	Hepatopancreas	*Lipid storage droplet protein*	168.34	2.22E-04

^a^Fold changes (Log2 ratio) in gene expression. Inf indicate gene was expressed in the estradiol group but not in the control group.

### Validation of transcriptomic sequencing by quantitative PCR analysis

**Fig 6:**
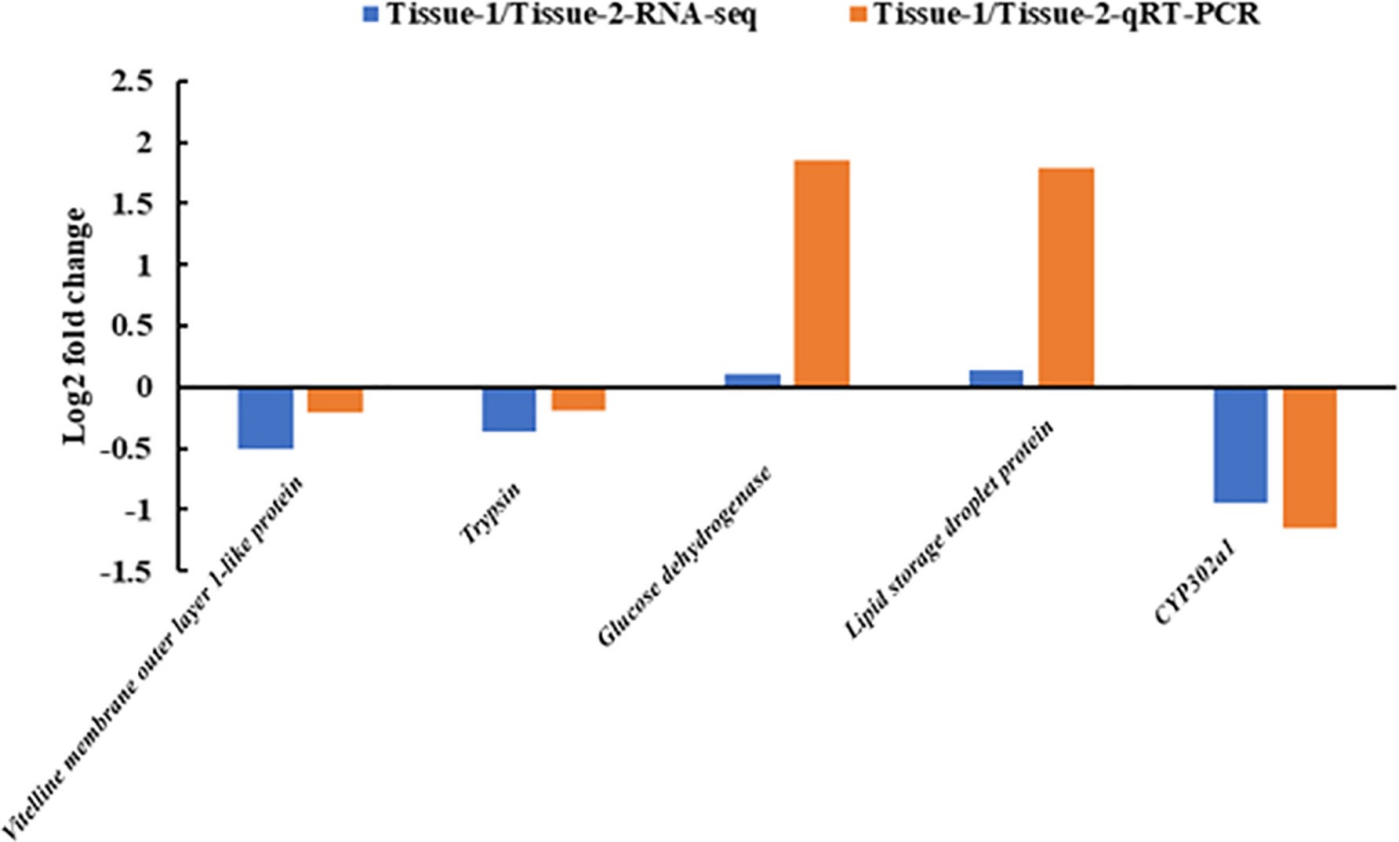
Comparison of gene expression patterns obtained using RNA-Seq and qRT-PCR. Notes: Log-fold changes are expressed as the ratio of gene expression after normalization to 18S. The 1 and 2 represent estradiol group and control group, respectively.

To confirm the sequencing results, 5 DEGs involved in ovarian development and nutrient metabolism were chosen for qRT-PCR analysis using the same RNA samples. These DEGs include *vitelline membrane outer layer 1-like protein*, *trypsin*, *glucose dehydrogenase*, *lipid storage droplet protein*, and *cytochrome P302a1*. The results showed that the expression of all genes can be detected, and the patterns of differential expression are consistent with the results from transcriptome data (**Fig 6**).

## Discussion

### Estradiol-responsive gene related to ovarian development

The endocrine regulation of ovarian development, involving multiple hormonal that secreted by different organs, has been studied in many crustacean species, including crayfish, shrimp, crab, lobsters [3, 36–38]. Different organs play different roles during the reproduction of crustacean, such as, ovary are the primary reproductive organs, whose normal development is crucial for reproduction [3, 39]. As the central organ for the absorption and storage of nutrients, hepatopancreas is also an important site for the synthesis of vitellogenin and metabolism of sex steroid hormones [34, 40–43]. Brain ganglion, eyestalk (X-organ-sinus gland complex) and mandibular organ are known to be the major endocrine organ that secrete many hormones involved in crustacean reproduction [3, 44]. This initial study describes the transcriptomic response of *P*. *trituberculatus* to short-term estradiol injection, and these unigenes could be used to reveal new insights into the estradiol regulation of ovarian development.

In the present study, a large number of key genes that may participate in the ovarian development were detected in the transcriptomic sequencing data. Vg is considered to be a biological marker of ovarian development, which is widely used in the evaluation of reproduction and endocrine disruption for both vertebrates and invertebrates [45–47]. However, estradiol injection did not significantly increase the expression of *Vg* in the transcriptome. This may be attributed to the time lag between the hormone induction and *Vg* expression. Similar results were also found in *Scylla paramamosain* [48]. Another gene associated with vitellogenesis, the transcript of *vitelline membrane outer layer 1-like protein* was regulated by estradiol injection, which is an important protein for developing oocytes and its major function is to avoid mixing of yolk and albumen [49].

Although estradiol did not significantly upregulate the gene expression related to vitellogenesis, the expression level of genes related to ovarian development were significantly altered by estradiol injection in various tissues of *P*. *trituberculatus*. Heat shock proteins are very conserved and involved in protein folding, degradation, and transportation, and their expression levels were regulated by sex steroid hormones [50, 51]. It has been proved that Heat shock protein 70 could be involved in estrogen nuclear-initiated steroid signaling as a molecular chaperone to negatively regulate Vg gene expression in *Metapenaeus ensis* [52, 53]. Therefore, the expression level of *Hsp70* was decreased may be related with the promotion of vitellogenesis by estradiol. *Vasa* is one of the important regulatory factors that determine the development of the reproductive system [54, 55]. In crustacean, vasa cDNAs have been reported in a variety of crustaceans, including *Litopenaeus vannamei* [56], *Fenneropenaeus chinensis* [57], *Macrobtachium rosenbergii* [58], *Parhyale hawaiensis* [59], *Scylla paramamosain* [60] and *Eriocheir sinensis* [61]. It has been proved that *vasa* are specifically expressed in gonads, and exogenous estradiol promotes the ovarian maturation by up-regulated the expression level of *vasa* in *Sparus aurata* [62]. Therefore, the up-regulation of *vasa* expression level in this study may be related to the positive regulation of estradiol on ovarian development.

The *wnt* signaling pathway is a conservative signaling network, which takes part in embryonic development, cell differentiation and proliferation, and the process of growth regulation [63]. As a signaling factor, Wnt5 plays various roles in vertebrate development [64–67]. Importantly, Wnt5 could coordinate with Wnt4 to initiate the meiosis for ovarian follicular growth in mammals [68]. The expression level of *Wnt5a ligand* and *Wnt5b* significantly increased after estradiol injection, indicating estradiol might regulate the ovarian development of *P*. *trituberculatus* by affecting the expression of Wnt signal pathway related genes. Further research is required to elucidate the roles of these genes in the reproductive process of crab. Progesterone is a hormone controlling the reproductive development, which not only stimulates yolk protein synthesis and ovarian maturation in *Penaeus vannamei* and *Scylla paramamosain*, but also stimulates pawning in *Metapenaeus ensis* [69–71]. It has also been reported that progesterone induces oocyte maturation by the activation of cyclin B [72]. In this study, two transcripts, encoding *cytoplasmic polyadenylation element binding protein* and *Cyclin B*, involved in regulation of progesterone on oocyte maturation pathway were found to be upregulated by estradiol injection. This indicated that an interaction between estradiol and progesterone-mediated oocyte maturation pathway might exist in crab.

Ovarian development is modulated by a variety of hormones, Thus, the expression of thirteen genes encoding hormones is also affected by estradiol in this study. Methyl farnesoate, a unepoxidated form of insect juvenile hormone III, is secreted by the mandibular organ in crustacean, and has obvious stimulatory function on Vg synthesis in various decapod species, including *Libinia emarginata*, *Cancer pagurus and Procambarus clarkii* [4, 21, 73, 74]. The biosynthetic pathway of methyl farnesoate has been well established in arthropods, which is divided into two steps [75]. In the first step, farnesyl pyrophosphate (FPP) is produced by the classical mevalonate pathway [76]. Then, the FPP is hydrolyzed to farnesol and oxidized to farnesoic acid that will be converted to methyl farnesoate via farnesoic acid O-methyl transferase [77]. However, little is known about its degradation in crustaceans. In insects, the juvenile hormone esterase, a carboxylesterase, is responsible for JH inactivation [78]. Previous studies showed that methyl farnesoate is metabolized to farnesoic acid in vitro by esterases that present in crustacean tissues [79]. In this study, four transcripts related to methyl farnesoate metabolism pathway were identified, including two *JHE*-*like carboxylesterase*, *Juvenile hormone esterase-like protein* and *Juvenile hormone acid methyltransferase*. Estradiol up-regulated the expression level of *JHE-like carboxylesterase 1*, *JHE-like carboxylesterase 2* and *Juvenile hormone esterase-like protein* in hepatopancreas and brain ganglion, indicating that estradiol promoted the methyl farnesoate inactivation in two tissues. However, estradiol reduced the transcript of *juvenile hormone acid methyltransferase* and *JHE-like carboxylesterase 1* in mandibular organ, which may indicate that the effect of estradiol on methyl farnesoate metabolism in mandibular organ is different from hepatopancreas and brain ganglion. As we know, Cytochrome P 302a1(CYP302a1) plays a critical role during ecdysteroid, another important reproductive hormone, biosynthesis process, that catalyzes the conversion of 5β-ketodiol to 20-hydroxyecdysone [80]. In the present study, estradiol reduced the transcript of *CYP302a1*, indicating that an increase in concentration of estradiol may lead to a decrease of ecdysone synthesis. Moreover, estrogen sulfotransferase (EST), the enzyme responsible for the sulfonation and inactivation of estrogens, plays an important role in estrogen homeostasis [81]. Previous studies have shown that the crustacean hepatopancreas is an important site for steroid hormone catabolism [72, 82]. The expression level of *EST* was up-regulated in hepatopancreas indicating that the crustaceans may have a self-regulation mechanism to metabolize and excrete the excess exogenous estradiol. Similar result was also found in other crab [6].

In crustaceans, crustacean hyperglycemic hormone family, a very important reproductive neuropeptides that mainly synthesized in the X-organ in the eyestalk, possess five types peptides, including crustacean hyperglycemic hormone, ion transport peptides, molting inhibitory hormone, vitellogenesis-inhibiting hormone, and mandibular organ-inhibiting hormone, which exhibit diversified physiological functions [83]. Previous studies showed that crustacean hyperglycemic hormone and molting inhibitory hormone promoted the process of vitellogenesis and ovarian development in decapod crustacean [17, 19]. Besides crustacean hyperglycemic hormone family, SIFamide, Neuropeptide F2 and C-type allatostatin were also secreted by eyestalk and cerebral ganglia. It has been reported that Neuropeptides may involve in stimulating food intake to supply energy for ovarian maturation in *Scylla paramamosain*, while allatostatins reduce the methyl farnesoate synthesis and hence repress ovarian maturation [84]. In this present study, estradiol injection significantly up-regulated the expression of *crustacean hyperglycemic hormone 1*, *molting inhibitory hormone*, *SIFamide* and *Neuropeptide F2*, while the expression of *C-type allatostatin* was significantly down-regulated. It can be speculated that estradiol regulates ovarian development by affecting the balance of neuropeptides in crustaceans.

### Estradiol-responsive gene related to nutrition metabolism

Based on previous studies, estradiol is involved in regulating metabolic processes [85, 86]. Therefore, further understanding the changes in genes associated with protein and lipid metabolism is essential in the transcriptome of *P*. *trituberculatus* to investigate its role on reproduction of crustacean. In this present study, three protease genes including *trypsin*, *trypsin-like serine proteinase* and *carboxypeptidase B*, were down-regulated by estradiol injection in the ovary of *P*. *trituberculatus*. Trypsin and carboxypeptidase are enzymes that cleave the peptide bond of different amino acid residue, while trypsin is reported to be involved in regulating the yolk degradation and yolk degradome activation [87, 88]. Proteins are one of the main constituents of yolk [2, 89]. It was speculated that estradiol indirectly promotes the process of yolk formation by reducing the expression of protein catabolism genes. Six candidate genes for lipid metabolism were up-regulated by estradiol in the hepatopancreas of *P*. *trituberculatus*, including *pancreatic triacylglycerol lipase-like*, *very low-density lipoprotein receptor*, *fatty acid elongase protein*, *lipid storage droplet protein* and *alcohol dehydrogenase*. In crustacean, lipid accumulating oocytes provides fuel for the biosynthetic processes of oogenesis and vitellogenesis and is later used by developing larvae [90]. Lipid storage droplets (LSDs) are organelles that accumulate lipid for both long- and short-term storage occurring in many animal cell types [91]. A recent study in *Penaeus monodon* indicated that the expression level of *LSD* significantly up-regulated during ovarian maturation [92, 93]. In this study, estradiol promoted the expression of *LSD* in hepatopancreas, indicating that estradiol can provide energy for ovarian development of *P*. *trituberculatus* by promoting the accumulation of LSD in the hepatopancreas.

Although above results have shown that exogenous estradiol had positive effects on genes relative to ovarian development of *P*. *trituberculatus*, the regulation mechanism of estradiol is largely unknown [5, 38, 94]. In vertebrate animals, it is well-known that cellular estrogen signaling was mediated primarily via the estrogen receptor (ER), a family of nuclear hormone receptor-type transcription factors [95]. Unfortunately, to date, the ER gene has not been functionally identified for any crustacean species, which may have been lost during the evolution of arthropods [96]. However, the estrogen related receptor (ERR) with high sequence homology with the vertebrate ER has been found in *P*. *trituberculatus* [97]. In this study, estradiol injection altered the transcription of ERR in five tissues of *P*. *trituberculatus* (S1 Table). Previous study has shown that ERR involved in regulating the gonadal development and cellular energy balance in vertebrate [98, 99]. Our previous results showed that the expression level of *ERR* was also altered when long-term injection of estradiol into *P*. *trituberculatus* [26]. In future studies, it is necessary to in-depth study the role of ERR in the regulation of estradiol in crustaceans.

## Conclusion

This study investigates the transcriptome sequencing of ovary, hepatopancreas, brain ganglion, eyestalk and mandibular organ of a swimming crab within a relatively short period after estradiol injection (24 h, 0.1μg g^-1^ crab weight). Through this study, some key genes in correlation with the reproduction and nutrition metabolism were significantly affected by estradiol. These results will serve as important resources for future experiments that further investigate the role and regulation of estradiol in *P*. *trituberculatus*. However, the function of estradiol-responsive gene in ovarian development and their cross-talking warrants further investigation.

## Supporting information

S1 Table The expression level of receptor and steroidogenic-related genes in five tissues. (XLSX)

## References

[1] Meusy J, Payen G. Female reproduction in malacostracan crustacea. Zoological Science. 1988; 5: 217–265. 10.1111/j.1096-3642.1988.tb01730.x

[2] Zmora N, Trant J, Chan S, Chung J. Vitellogenin and its messenger RNA during ovarian development in the female blue crab, *Callinectes sapidus*: gene expression, synthesis, transport, and cleavage. Biology of Reproduction. 2007; 77(1): 138–146. 10.1095/biolreprod.106.055483 . 17409377

[3] Nagaraju G. Reproductive regulators in decapod crustaceans: an overview. Journal of Experimental Biology. 2011; 214(1): 3–16. 10.1242/jeb.047183 . 21147963

[4] Rodriguez E, Medesani D, Greco L, Fingerman M. Effects of some steroids and other compounds on ovarian growth of the red swamp crayfish, *Procambarus clarkii*, during early vitellogenesis. Journal of Experimental Zoology. 2002; 292(1): 82–87. 10.1002/jez.1144 . 11754024

[5] Yano I, Hoshino R. Effects of 17 beta-estradiol on the vitellogenin synthesis and oocyte development in the ovary of kuruma prawn (*Marsupenaeus japonicus*). Comparative Biochemistry and Physiology A-Molecular & Integrative Physiology. 2006; 144(1): 18–23. 10.1016/j.cbpa.2006.01.026 . 16545975

[6] Shen B, Yang X, Wu X, Cheng Y, Tang B. The effects of exogenous 17β-estradiol on ovary development and on the level of endogenous 17β-estradiol in *Eriocheir sinensis*. Journal of Shanghai Ocean University. 2010;19: 289–95 (In Chinese with English abstract).

[7] Koskela R, Greenwood J, Rothlisberg P. The influence of prostaglandin estradiol and the steroid hormones, 17α-hydroxyprogesterone and 17β-estradiol on moulting and ovarian development in the tiger prawn, *Penaeus esculentus* Haswell, 1879 (Crustacea: Decapoda). Comparative Biochemistry and Physiology Part A: Physiology. 1992; 101: 295–299. 10.1016/0300-9629(92)90535-X

[8] Tsukimura B, Bender J, Linder C. Development of an anti-vitellin ELISA for the assessment of reproduction in the ridgeback shrimp, *Sicyonia ingentis*. Comparative Biochemistry and Physiology Part A: Molecular and Integrative Physiology. 2000; 127(2): 215–24. 10.1016/s1095-6433(00)00255-5 . 11064288

[9] Tiu S, Hui J, He J, Tobe S, Chan S. Characterization of vitellogenin in the shrimp *Metapenaeus ensis*: expression studies and hormonal regulation of MeVg1 transcription in vitro. Molecular Reproduction and Development. 2006; 73(4): 424–36. 10.1002/mrd.20433 . 16425293

[10] Liu M, Pan J, Liu Z, Cheng Y, Gong J, Wu X. Effect of estradiol on vitellogenesis and oocyte development of female swimming crab, *Portunus trituberculatus*. Aquaculture. 2018; 486: 240–5. 10.1016/j.aquaculture.2017.12.034

[11] Quinitio E, Hara A, Yamauchi K, Nakao S. Changes in the steroid hormone and vitellogenin levels during the gametogenic cycle of the giant tiger shrimp, *Penaeus monodon*. Comparative Biochemistry and Physiology Part C: Pharmacology, Toxicology and Endocrinology. 1994; 109: 21–6. 10.1016/0742-8413(94)00044-B

[12] Martins J, Ribeiro K, Rangel-Figueiredo T, Coimbra J. Reproductive cycle, ovarian development, and vertebrate-type steroids profile in the freshwater prawn *Macrobrachium rosenbergii*. Journal of Crustacean Biology. 2007; 27(2): 220–8. 10.1651/c-2597.1

[13] Huang H, Ye H, Han S, Wang G. Profiles of gonadotropins and steroid hormone-like substances in the hemolymph of mud crab *Scylla paramamosain* during the reproduction cycle. Marine and Freshwater Behaviour and Physiology. 2009; 42(4): 297–305. 10.1080/10236240903174792

[14] MardisE. The impact of next-generation sequencing technology on genetics. Trends in Genetics. 2008; 24(3): 133–41. 10.1016/j.tig.2007.12.007 .18262675

[15] Subramoniam T. Crustacean ecdysteriods in reproduction and embryogenesis. Comparative Biochemistry and Physiology C-Pharmacology Toxicology & Endocrinology. 2000; 125(2): 135–56. 10.1016/s0742-8413(99)00098-5 . 11790337

[16] Ye H, Ai C, Huang H, Li S. Advances on reproductive physiology of crabs. Journal of Xiamen University (Natural Science). 2006; 45: 170–175 (In Chinese with English abstract). 10.3321/j.issn:0438-0479.2006.z2.024

[17] De Kleijn D, Janssen K, Waddy S, Hegeman R, Lai W, Martens G, et al Expression of the crustacean hyperglycaemic hormones and the gonad-inhibiting hormone during the reproductive cycle of the female American lobster *Homarus americanus*. Journal of Endocrinology. 1998; 156(2): 291–298. 10.1677/joe.0.1560291 . 9518875

[18] Gu P, Tobe S, Chow B, Chu K, He J, Chan S. Characterization of an additional molt inhibiting hormone-like neuropeptide from the shrimp *Metapenaeus ensis*. Peptides. 2002; 23(11): 1875–83. 10.1016/s0196-9781(02)00178-x . 12431725

[19] Zmora N, Trant J, Zohar Y, Chung J. Molt-inhibiting hormone stimulates vitellogenesis at advanced ovarian developmental stages in the female blue crab, *Callinectes sapidus* 1: an ovarian stage dependent involvement. Saline Systems. 2009; 5(1): 7 10.1186/1746-1448-5-7 19583852 PMC2715418

[20] Chen Y, Fan H, Hsieh S, Kuo C. Physiological involvement of DA in ovarian development of the freshwater giant prawn, *Macrobrachium rosenbergii*. Aquaculture. 2003; 228(1–4): 383–395. 10.1016/s0044-8486(03)00324-7

[21] Laufer H, Albrecht K. Metabolism of methyl farnesoate in vitro by peripheral tissues of the spider crab, *Libinia emarginata* (Decapoda). Advances in Invertebrate Reproduction. 1990; 5: 217–222.

[22] Gong J, Ye H, Xie Y, Yang Y, Huang H, Li S, et al Ecdysone receptor in the mud crab *Scylla paramamosain*: a possible role in promoting ovarian development. Journal of Endocrinology. 2015; 224(3): 273–287. 10.1530/JOE-14-0526 . 25563354

[23] Hamasaki K, Fukunaga K, Kitada S. Batch fecundity of the swimming crab *Portunus* trituberculatus (Brachyura: Portunidae). Aquaculture. 2006; 253(1–4): 359–365. 10.1016/j.aquaculture.2005.08.002

[24] Kim D, Kim S, Choi J, Kim B, Seo H, Jang I. The effects of manipulating water temperature, photoperiod, and eyestalk ablation on gonad maturation of the swimming crab, *Portunus trituberculatus*. Crustaceana. 2010; 83(2): 129–141. 10.1163/001121609x12591347509248

[25] Liu M, Wu X, Pan J, Cheng Y. Immunolocalization and changes in 17β-estradiol in *Portunus trituberculatus* during ovarian development. Journal of Fishery Sciences of China. 2017; 24: 239–47 (In Chinese with English abstract). 10.3724/SP.J.1118.2017.16136

[26] Lu Y, Liu M, Gong J, Cheng Y, Wu X. Effect of exogenous estrogen on the ovarian development and gene expression in the female swimming crab *Portunus trituberculatus* (Miers, 1876) (Decapoda: Brachyura: Portunidae). Journal of Crustacean Biology. 2018; 38(3): 367–373. 10.1093/jcbol/ruy013

[27] Pan G, Hou W, Wu X, Wu R, Zhang N, Long X, et al Effects of water temperature and single crab basket culture on ovarian development and tissue proximate composition of female *Portunus trituberculatus*. Marine Fisheries. 2015; 37: 550–556. 10.3969/j.issn.1004-2490.2015.06.010

[28] Coccia E, De Lisa E, Di Cristo C, Di Cosmo A, Paolucci M. Effects of estradiol and progesterone on the reproduction of the freshwater crayfish *Cherax albidus*. Biological Bulletin. 2010; 218(1): 36–47. 10.1086/BBLv218n1p36 . 20203252

[29] Grabherr M, Haas B, Yassour M, Levin J, Thompson D, Amit I, et al Full-length transcriptome assembly from RNA-Seq data without a reference genome. Nature Biotechnology. 2011; 29(7): 644–652. 10.1038/nbt.1883 . 21572440 PMC3571712

[30] Ashburner M, Ball C, Blake J, Botstein D, Butler H, Cherry J, et al Gene Ontology: tool for the unification of biology. Nature Genetics. 2000; 25(1): 25–29. 10.1038/75556 . 10802651 PMC3037419

[31] Kanehisa M, Goto S, Kawashima S, Okuno Y, Hattori M. The KEGG resource for deciphering the genome. Nucleic Acids Research. 2004; 32: D277–D80. 10.1093/nar/gkh063 . 14681412 PMC308797

[32] Powell S, Forslund K, Szklarczyk D, Trachana K, Roth A, Huerta-Cepas J, et al eggNOG v4.0: nested orthology inference across 3686 organisms. Nucleic Acids Research. 2014; 42(D1): 231–239. 10.1093/nar/gkt1253 . 24297252 PMC3964997

[33] Li B, Dewey C. RSEM: accurate transcript quantification from RNA-Seq data with or without a reference genome. BMC Bioinformatics. 2011; 12: 16 10.1186/1471-2105-12-16 . 21816040 PMC3163565

[34] Yang F, Xu H, Dai Z, Yang W. Molecular characterization and expression analysis of vitellogenin in the marine crab *Portunus trituberculatus*. Comparative Biochemistry and Physiology Part B: Biochemistry & Molecular Biology. 2005; 142(4): 456–464. 10.1016/j.cbpb.2005.09.011 . 16257250

[35] Livak K, Schmittgen T. Analysis of relative gene expression data using real-time quantitative PCR and the 2(T)(-Delta Delta C) method. Methods. 2001; 25(4): 402–408. 10.1006/meth.2001.1262 . 11846609

[36] Fingerman M. Crustacean endocrinology: A retrospective, prospective, and introspective analysis. Physiological Zoology. 1997; 70(3): 257–269. 10.1086/639593 . 9231399

[37] Chang E, Chang S, Mulder E. Hormones in the lives of crustaceans: An overview. American Zoologist. 2001; 41(5):1090–1097. 10.1668/0003-1569(2001)041[1090:Hitloc]2.0.Co;2 WOS:000174175500005.

[38] Subramoniam T. Chapter 9—Endocrine Regulation of Vitellogenesis In: Subramoniam T, editor. Sexual Biology and Reproduction in Crustaceans: Academic Press; 2017 p. 231–67.

[39] Wang Z, Sun L, Guan W, Zhou C, Tang B, Cheng Y, et al De novo transcriptome sequencing and analysis of male and female swimming crab (*Portunus trituberculatus*) reproductive systems during mating embrace (stage II). Bmc Genetics. 2018; 19(1): 3 10.1186/s12863-017-0592-5 . 29298661 PMC5753516

[40] Vogt G, Stöcker W, Storch V, Zwilling R. Biosynthesis of astacus, protease, a digestive enzyme from crayfish. Histochemistry. 1989; 91(5): 373–381. 10.1007/bf00493824 2656593

[41] Vogt G. Life-cycle and functional cytology of the hepatopancreatic cells of *Astacus astacus* (Crustacea, Decapoda). Zoomorphology. 1994; 114: 83–101. 10.1007/BF00396642

[42] Cheng Y, Du N, Lai W. The lipid accumulation during the stages of the ovarian fast maturation and their effect on the spawning of *Eriocheir sinensis*. Journal of Fisheries of China. 2000; 24: 113–119 (In Chinese with English abstract). 10.3321/j.issn:1000-0615.2000.02.004

[43] Summavielle T, Monteiro P, Reis-Henriques M, Coimbra J. In vitro metabolism of steroid hormones by ovary and hepatopancreas of the crustacean Penaeid shrimp *Marsupenaeus japonicus*. Scientia Marina. 2003; 67(3): 299–306. 10.3989/scimar.2003.67n3299

[44] Suwansa-ard S, Thongbuakaew T, Wang T, Zhao M, Elizur A, Hanna P, et al In silico neuropeptidome of female *Macrobrachium rosenbergii* based on transcriptome and peptide mining of eyestalk, central nervous system and ovary. Plos One. 2015; 10(5): e0123848 10.1371/journal.pone.0123848 . 26023789 PMC4449106

[45] Sumpter J, Jobling S. Vitellogenesis as a biomarker for estrogenic contamination of the aquatic environment. Environmental Health Perspectives. 1995; 103: 173–178. 10.2307/3432529 . 8593867 PMC1518861

[46] Marin M, Matozzo V. Vitellogenin induction as a biomarker of exposure to estrogenic compounds in aquatic environments. Marine Pollution Bulletin. 2004; 48(9–10): 835–839. 10.1016/j.marpolbul.2004.02.037 . 15111030

[47] Porte C, Janer G, Lorusso L, Ortiz-Zarragoitia M, Cajaraville M, Fossi M, et al Endocrine disruptors in marine organisms: Approaches and perspectives. Comparative Biochemistry and Physiology Part C-Toxicology & Pharmacology. 2006; 143(3): 303–315. 10.1016/j.cbpc.2006.03.004 . 16723279

[48] Gong J, Huang C, Shu L, Bao C, Huang H, Ye H, et al The retinoid X receptor from mud crab: new insights into its roles in ovarian development and related signaling pathway. Scientific Reports. 2016; 6: 23654 10.1038/srep23654 . 27009370 PMC4806290

[49] Sricharoen S, Kim J, Tunkijjanukij S, Soderhall I. Exocytosis and proteomic analysis of the vesicle content of granular hemocytes from a crayfish. Developmental and Comparative Immunology. 2005; 29(12): 1017–1031. 10.1016/j.dci.2005.03.010 . 15975654

[50] Mayer M, Bukau B. Hsp70 chaperones: Cellular functions and molecular mechanism. Cellular and Molecular Life Sciences. 2005; 62(6): 670–684. 10.1007/s00018-004-4464-6 . 15770419 PMC2773841

[51] Romani W, Russ D. Acute effects of sex-specific sex hormones on heat shock proteins in fast muscle of male and female rats. European Journal of Applied Physiology. 2013;113(10): 2503–2510. 10.1007/s00421-013-2686-8 . 23821238

[52] Dhamad A, Zhou Z, Zhou J, Du Y. Systematic proteomic identification of the Heat Shock Proteins (Hsp) that interact with estrogen receptor alpha (ER alpha) and biochemical characterization of the ER alpha-Hsp70 interaction. Plos One. 2016; 11(8): e0160312 10.1371/journal.pone.0160312 . 27483141 PMC4970746

[53] Chan S, He J, Chu K, Sun C. The shrimp heat shock cognate 70 functions as a negative regulator in vitellogenin gene expression. Biology of Reproduction. 2014; 91(1): 14 10.1095/biolreprod.113.117200 . 24790159

[54] Lasko P, Ashburner M. The product of the Drosophila gene vasa is very similar to eukaryotic initiation factor-4A. Nature. 1988; 335: 611–617. 10.1038/335611a0 3140040

[55] Hay B, Jan L, Jan Y. A protein component of Drosophila polar granules is encoded by vasa and has extensive sequence similarity to ATP-dependent helicases. Cell. 1988; 55: 577–587. 10.1016/0092-8674(88)90216-4 3052853

[56] Aflalo E, Bakhrat A, Raviv S, Harari D, Sagi A, Abdu U. Characterization of a Vasa-like gene from the Pacific white shrimp *Litopenaeus vannamei* and its expression during oogenesis. Molecular Reproduction and Development. 2007; 74(2): 172–177. 10.1002/mrd.20622 . 16955407

[57] Zhou Q, Shao M, Qin Z, Kang Q, Zhang Z. Cloning, characterization, and expression analysis of the DEAD-box family genes, Fc-vasa and Fc-PL10a, in Chinese shrimp (*Fenneropenaeus chinensis*). Chinese Journal of Oceanology and Limnology. 2010; 28: 37–45 (In Chinese with English abstract). 10.1007/s00343-010-9231-y

[58] Nakkrasae L, Damrongphol P. A vasa-like gene in the giant freshwater prawn, *Macrobrachium rosenbergii*. Molecular Reproduction and Development. 2007; 74(7): 835–842. 10.1002/mrd.20680 . 17186538

[59] Extavour C, Akam M. Mechanisms of germ cell specification across the metazoans: epigenesis and preformation. Development. 2003; 130(24): 5869–5884. 10.1242/dev.00804 . 14597570

[60] Wang Y, Chen Y, Han K, Zou Z, Zhang Z. A vasa gene from green mud crab *Scylla paramamosain* and its expression during gonadal development and gametogenesis. Molecular Biology Reports. 2012; 39(4): 4327–4335. 10.1007/s11033-011-1220-5 . 21842219

[61] Wang Q, Fang D, Sun J, Wang Y, Wang J, Liu L. Characterization of the vasa gene in the Chinese mitten crab *Eriocheir sinensis*: a germ line molecular marker. Journal of Insect Physiology. 2012;58(7):960–5. 10.1016/j.jinsphys.2012.04.012 . 22562064

[62] Cardinali M, Gioacchini G, Candiani S, Pestarino M, Yoshizaki G, Carnevali O. Hormonal regulation of vasa-like messenger RNA expression in the ovary of the marine teleost *Sparus aurata*. Biology of Reproduction. 2004; 70(3): 737–743. 10.1095/biolreprod.103.021428 . 14613903

[63] Gao J, Wang X, Zou Z, Jia X, Wang Y, Zhang Z. Transcriptome analysis of the differences in gene expression between testis and ovary in green mud crab (*Scylla paramamosain*). Bmc Genomics. 2014; 15(1): 585 10.1186/1471-2164-15-585 . 25015001 PMC4124137

[64] Prathibha Y, Senthilkumaran B. Expression of wnt4/5 during reproductive cycle of catfish and wnt5 promoter analysis. Journal of Endocrinology. 2017; 232(1): 1–13. 10.1530/JOE-16-0104 . 27875264

[65] Kim H, Schleiffarth J, Jessurun J, Sumanas S, Petryk A, Lin S, et al Wnt5 signaling in vertebrate pancreas development. Bmc Biology. 2005; 3(1): 23 10.1186/1741-7007-3-23 . 16246260 PMC1276788

[66] Huang L, Xiao A, Choi S, Kan Q, Zhou W, Chacon-Heszele M, et al Wnt5a is necessary for normal kidney development in zebrafish and mice. Nephron Experimental Nephrology. 2014; 128(1–2): 80–88. 10.1159/000368411 . 25412793 PMC4382204

[67] Kilian B, Mansukoski H, Barbosa F, Ulrich F, Tada M, Heisenberg C. The role of Ppt/Wnt5 in regulating cell shape and movement during zebrafish gastrulation. Mechanisms of Development. 2003; 120(4): 467–476. 10.1016/s0925-4773(03)00004-2 12676324

[68] Naillat F, Prunskaite-Hyyrylainen R, Pietila I, Sormunen R, Jokela T, Shan J, et al Wnt4/5a signalling coordinates cell adhesion and entry into meiosis during presumptive ovarian follicle development. Human Molecular Genetics. 2010; 19(8): 1539–1550. 10.1093/hmg/ddq027 . 20106871

[69] Scott Q. Yolk synthesis in the marine shrimp, *Penaeus vannamei*. American Zoologist. 2001; 41: 458–464. 10.2307/3884476

[70] Ye H, Song P, Ma J, Huang H, Wang G. Changes in progesterone levels and distribution of progesterone receptor during vitellogenesis in the female mud crab (*Scylla paramamosain*). Marine and Freshwater Behaviour and Physiology. 2010; 43(1): 25–35. 10.1080/10236241003654113

[71] Yano I. Induced ovarian maturation and spawning in greasyback shrimp, *Metapenaeus ensis*, by progesterone. Aquaculture. 1985; 47: 223–229. 10.1016/0044-8486(85)90068-7

[72] Uawisetwathana U, Leelatanawit R, Klanchui A, Prommoon J, Klinbunga S, Karoonuthaisiri N. Insights into eyestalk ablation mechanism to induce ovarian maturation in the Black Tiger Shrimp. Plos One. 2011; 6(9): e24427 10.1371/journal.pone.0024427 . 21915325 PMC3168472

[73] Wainwright G, Prescott M, Rees H, Webster S. Mass spectrometric determination of methyl farnesoate profiles and correlation with ovarian development in the edible crab, *Cancer pagurus*. Journal of Mass Spectrometry. 1996; 31(12): 1338–1344. 10.1002/(sici)1096-9888(199612)31:12<1338::Aid-jms428>3.0.Co;2-a

[74] Nagaraju G. Is methyl farnesoate a crustacean hormone? Aquaculture. 2007; 272(1–4): 39–54. 10.1016/j.aquacutture.2007.05.014

[75] Borst D, Ogan J, Tsukimura B, Claerhout T, Holford K. Regulation of the crustacean mandibular organ. American Zoologist. 2001; 41(3): 430–441. 10.1668/0003-1569(2001)041[0430:Rotcmo]2.0.Co;2

[76] Goldstein J, Brown M. Regulation of the mevalonate pathway. Nature. 1990; 343: 425–430. 10.1038/343425a0 1967820

[77] Holford K, Edwards K, Bendena W, Tobe S, Wang Z, Borst D. Purification and characterization of a mandibular organ protein from the American lobster, *Homarus americanus*: a putative farnesoic acid O-methyltransferase. Insect Biochemistry and Molecular Biology. 2004; 34(8): 785–798. 10.1016/j.ibmb.2004.04.003 . 15262283

[78] Kontogiannatos D, Swevers L, Maenaka K, Park E, Iatrou K, Kourti A. Functional characterization of a juvenile hormone esterase related gene in the moth *Sesamia nonagrioides* through RNA Interference. Plos One. 2013; 8(9): e73834 10.1371/journal.pone.0073834 . 24040087 PMC3770702

[79] Tobe S, Young D, Khoo H, Baker F. Farnesoic acid as a major product of release from crustacean mandibular organs in vitro. Journal of Experimental Zoology.1989; 249(2): 165–171. 10.1002/jez.1402490208

[80] Niwa R, Sakudoh T, Namiki T, Saida K, Fujimoto Y, Kataoka H. The ecdysteroidogenic P450 Cyp302a1/disembodied from the silkworm, *Bombyx mori*, is transcriptionally regulated by prothoracicotropic hormone. Insect Molecular Biology. 2005; 14(5): 563–571. 10.1111/j.1365-2583.2005.00587.x . 16164612

[81] Gao J, He J, Shi X, Stefanovic-Racic M, Xu M, O'Doherty R, et al Sex-specific effect of estrogen sulfotransferase on mouse models of Type 2 diabetes. Diabetes. 2012; 61(6): 1543–1551. 10.2337/db11-1152 . 22438574 PMC3357292

[82] Swevers L, Lambert J, Loof A. Metabolism of vertebrate-type steroids by tissues of three crustacean species. Comparative Biochemistry and Physiology Part B: Comparative Biochemistry. 1991; 99: 35–41. 10.1016/0305-0491(91)90004-W

[83] Webster S, Keller R, Dircksen H. The CHH-superfamily of multifunctional peptide hormones controlling crustacean metabolism, osmoregulation, moulting, and reproduction. General and Comparative Endocrinology. 2012;175(2):217–33. 10.1016/j.ygcen.2011.11.035 . 22146796

[84] Bao C, Yang Y, Huang H, Ye H. Neuropeptides in the cerebral ganglia of the mud crab, *Scylla paramamosain*: transcriptomic analysis and expression profiles during vitellogenesis. Scientific Reports. 2015; 5: 17055 10.1038/srep17055 . 26592767 PMC4655400

[85] Hussain Y, Ding Q, Connelly P, Brunt J, Ban M, McIntyre A, et al G-Protein estrogen receptor as a regulator of low-density lipoprotein cholesterol metabolism cellular and population genetic studies. Arteriosclerosis Thrombosis and Vascular Biology. 2015; 35(1): 213–221. 10.1161/atvbaha.114.304326 . 25395619

[86] Oosthuyse T, Bosch A. Oestrogen's regulation of fat metabolism during exercise and gender specific effects. Current Opinion in Pharmacology. 2012; 12(3): 363–371. 10.1016/j.coph.2012.02.008 . 22398320

[87] Skern-Mauritzen R, Frost P, Dalvin S, Kvamme B, Sommerset I, Nilsen F. A trypsin-like protease with apparent dual function in early *Lepeophtheirus salmonis* (Kroyer)development. Bmc Molecular Biology. 2009; 10(1): 1–11. 10.1186/1471-2199-10-44 . 19439101 PMC2689223

[88] Wu J, Min R, Wu M, Chen W. Research progresses on the carboxypeptidase. Journal of Food Science and Biotechnology. 2012; 31: 793–801 (In Chinese with English abstract). 10.3969/j.issn.1673-1689.2012.08.002

[89] Chen L, Jiang H, Zhou Z, Li K, Li K, Deng G, et al Purification of vitellin from the ovary of Chinese mitten-handed crab (*Eriocheir sinensis*) and development of an antivitellin ELISA. Comparative Biochemistry and Physiology Part B: Biochemistry & Molecular Biology. 2004; 138(3): 305–311. 10.1016/j.cbpc.2004.04.012 . 15253879

[90] Harrison K. The role of nutrition in maturation, reproduction and embryonic development of decapod crustaceans: a review. Journal of Shellfish Research. 1990; 9: 1–28.

[91] Murphy D. The biogenesis and functions of lipid bodies in animals, plants and microorganisms. Progress in Lipid Research. 2001; 40(5): 325–438. 10.1016/s0163-7827(01)00013-3 . 11470496

[92] Sittikankaew K, Hiransuchalert R, Yocawibun P, Yamano K, Klinbunga S. Identification, characterization and expression of adipose differentiation-related protein (ADRP) gene and protein in ovaries of the giant tiger shrimp *Penaeus monodon*. Aquaculture. 2010; 308: S91–S9. 10.1016/j.aquaculture.2010.06.039

[93] Brady P, Elizur A, Cummins S, Ngyuen N, Williams R, Knibb W. Differential expression microarrays reveal candidate genes potentially associated with reproductive dysfunction of captive-reared prawn *Penaeus monodon*. Aquaculture. 2013; 400: 14–28. 10.1016/j.aquaculture.2013.02.038

[94] Merlin J, Mohanlal D, Balasubramanian C, Sherly T, Subramoniam T, Syamadayal J, et al Induction of vitellogenesis and reproductive maturation in tiger shrimp, *Penaeus monodon* by 17ß-estradiol and 17α-hydroxyprogesterone: in vivo and in vitro studies. Invertebrate Reproduction & Development. 2015; 59(3): 166–175. 10.1080/07924259.2015.1051192

[95] Qiu S, Vazquez J, Boulger E, Liu H, Xue P, Hussain M, et al Hepatic estrogen receptor α is critical for regulation of gluconeogenesis and lipid metabolism in males. Scientific Reports. 2017; 7(1): 1661 10.1038/s41598-017-01937-4 . 28490809 PMC5431852

[96] Thornton J, Need E, Crews D. Resurrecting the ancestral steroid receptor: ancient origin of estrogen signaling. Science. 2003; 301(5640): 1714–1717. 10.1126/science.1086185 . 14500980

[97] Lu Y, Wu X, Pan G, Wang W, Hou W, Cheng Y. Expression analysis of PtERR during the molting cycle in *Portunus trituberculatus*. Journal of Shanghai Ocean University. 2016; 25(3): 321–328 (In Chinese with English abstract). 10.12024/jsou.20150801528

[98] Giguère V. Orphan nuclear receptors: from gene to function. Endocrine Reviews. 1999; 20(5): 689–725. 10.1210/edrv.20.5.0378 10529899

[99] Tarrant A, Greytak S, Callard G, Hahn M. Estrogen receptor-related receptors in the killifish *Fundulus heteroclitus*: diversity, expression, and estrogen responsiveness. Journal of Molecular Endocrinology. 2006; 37(1): 105–120. 10.1677/jme.1.01976 . 16901928

